# Construction of a prognostic model for endometrial cancer related to programmed cell death using WGCNA and machine learning algorithms

**DOI:** 10.3389/fimmu.2025.1564407

**Published:** 2025-05-20

**Authors:** Weicheng Pan, Jinlian Cheng, Shanshan Lin, Qianxi Li, Yuanyuan Liang, Huiying Li, Xianxian Nong, Huizhen Nong

**Affiliations:** ^1^ Department of Obstetrics and Gynecology, Wuming Hospital of Guangxi Medical University, Nanning, Guangxi, China; ^2^ Department of Obstetrics and Gynecology, The First Affiliated Hospital of Guangxi Medical University, Nanning, Guangxi, China

**Keywords:** endometrial cancer, programmed cell death, machine learning, WGCNA, prognostic model

## Abstract

**Background:**

Programmed cell death (PCD) refers to a regulated and active process of cellular demise, initiated by specific biological signals. PCD plays a crucial role in the development, progression, and drug resistance of uterine corpus endometrial carcinoma (UCEC), making the exploration of its relationship with UCEC prognosis highly clinically relevant.

**Methods:**

Data from UCEC patients and control cohorts were obtained from The Cancer Genome Atlas (TCGA) database. Differentially expressed genes (DEGs) were identified and subsequently intersected with a PCD gene set to discern PCD-related differentially expressed genes (PCD-DEGs). To isolate core prognostic PCD-DEGs, methods including consistency clustering analysis, weighted gene co-expression network analysis (WGCNA), univariate Cox regression analysis, and five machine learning techniques for dimensionality reduction were utilized. Validation of three core prognostic PCD-DEGs was conducted using RT-qPCR, and these genes were used to develop a prognostic model. Additionally, an analysis of drug sensitivity was performed.

**Results:**

Consistency clustering analysis revealed significant differences in prognosis and tumor microenvironment among subtypes, strongly associated with various immune subtypes. The three core prognostic PCD-DEGs identified—SRPX, NT5E, and ATP6V1C2—were instrumental in constructing the lasso prognostic model and nomogram. Receiver Operating Characteristic (ROC) curve analysis confirmed the model’s strong prognostic performance and clinical applicability. The high-risk group exhibited lower tumor mutation frequencies, a higher propensity for immune escape, reduced response to immune therapy, and potential benefits from potent chemotherapy drugs.

**Conclusion:**

This study developed a prognostic model related to PCD for UCEC using comprehensive bioinformatics analyses. The model demonstrates robust predictive performance and holds significant potential for clinical application, thereby facilitating precise stratification and personalized treatment of UCEC patients.

## 1 Introduction

UCEC ranks as one of the leading malignant neoplasms in the female reproductive system, with a rising incidence rate globally (1). The integration of surgical intervention with radiotherapy and chemotherapy can enhance prognosis for certain patients; however, prognostic outcomes show considerable variability among individuals. This variability is attributed to the inherent heterogeneity of UCEC and the diverse array of clinical and pathological characteristics (2, 3). Currently, traditional prognostic evaluation methods primarily rely on tumor staging, grading, and clinical pathological parameters. However, these methods are insufficient in comprehensively reflecting the molecular biological characteristics of UCEC, thereby limiting their ability to guide personalized treatment for patients (4). Consequently, the development of clinical prognostic models based on molecular biomarkers, which can accurately predict patient survival risks and provide reliable evidence for individualized treatment plans, is a current research hotspot (5–7).

PCD constitutes a cellular demise process that is meticulously regulated by intrinsic mechanisms and includes various forms, such as apoptosis, pyroptosis, ferroptosis, and autophagic cell death (8, 9). Within the context of tumorigenesis and progression, PCD plays a dualistic role. It functions as a tumor suppressor by inhibiting aberrant cell proliferation; conversely, tumor cells may manipulate PCD pathways to evade immune surveillance, thereby facilitating malignant progression (8, 10). Recent studies have shown that genes associated with PCD exhibit significant diagnostic and prognostic value in various cancers (11–13). However, in endometrial cancer, the functions, expression patterns, and clinical significance of PCD-related genes remain underexplored, and there is a lack of systematic research.

Thus, thoroughly analyzing the expression patterns and clinical importance of PCD-related genes in UCEC will aid in understanding the tumor’s molecular mechanisms and highlight their potential value in prognosis and diagnosis. Establishing a clinical prognostic model related to PCD for UCEC provides valuable guidance for clinical decision-making in UCEC.

## 2 Materials and methods

### 2.1 Data collection

**Figure 1:**
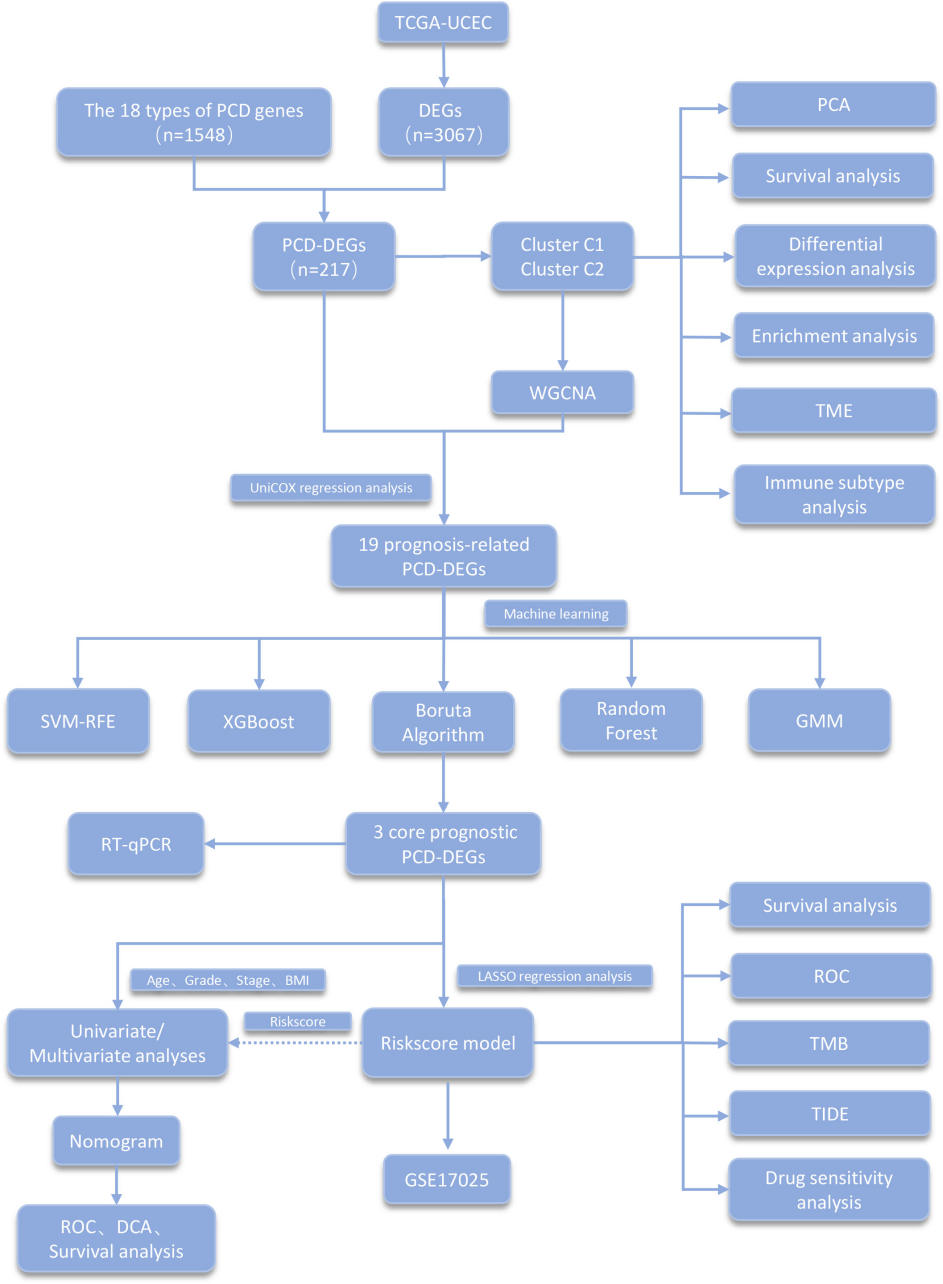
Main flowchart of study design.

**Figure 1** presents a diagram illustrating the framework of this study. We obtained the expression and clinical data of UCEC from The Cancer Genome Atlas (TCGA) database. The clinical dataset for TCGA-UCEC is organized in **Supplementary Table S1**. The compilation of genes related to PCD utilized resources including the MSigDB, GeneCards, the Kyoto Encyclopedia of Genes and Genomes (KEGG), and extensive literature reviews (14). In total, 18 types of PCD were identified, which include Immunogenic Cell Death, Pyroptosis, Paraptosis, Entosis, Cuproptosis, Parthanatos, NETosis, Lysosome-dependent Cell Death, Ferroptosis, Alkaliptosis, Apoptosis, Oxeiptosis, Netotic Cell Death, Anoikis, Autophagy, Methuosis, Entotic Cell Death, and Necroptosis. After removing duplicate entries, 1,548 genes associated with PCD were cataloged (**Supplementary Table S2**).

From the Department of Obstetrics and Gynecology at Wuming Hospital, Guangxi Medical University, 20 samples of UCEC tissue and 20 samples of control tissue were collected. The staging of UCEC was assessed according to the 2009 FIGO guidelines, with evaluations conducted by experienced pathologists. For the control group, endometrial tissues were selected from patients undergoing hysterectomies without any underlying endometrial conditions. Clinical data for the UCEC patients are presented in **Table 1**. This research was conducted in accordance with the guidelines of the Declaration of Helsinki and received ethical approval from the Ethics Committee of Wuming Hospital, Guangxi Medical University (approval number: WM-2024(237)). All participants provided written informed consent prior to undergoing surgery.

**Table 1:** The clinical characteristics of the 20 UCEC patients used for validation.

Clinical parameters		N
Age	<60	13
	≥60	7
Grade	G1	11
	G2	8
	G3	1
Stage	IA	18
	IB	2
	II-IV	0
Lymphovascular space invasion	Yes	2
	No	18

### 2.2 Differential expression analysis and consensus clustering

The TCGA-UCEC dataset was subjected to differential expression analysis employing the ‘limma’ package, adhering to a filtering criterion of an absolute log2 fold change (|log2 fold change|) of at least 1.5 and an adjusted p-value of less than 0.05. To identify PCD-DEGs, log2 normalization was applied to the identified DEGs. Subsequently, an intersection of these genes with those related to PCD was performed.

Consensus clustering was conducted on 543 UCEC samples, utilizing the expression data of PCD-DEGs to delineate potential molecular subtypes. In this analysis, the maximum number of clusters was established at nine (maxK = 9). The K-means algorithm, using Euclidean distance as the metric, was employed. The algorithm was iterated 50 times (reps = 50), randomly selecting 80% of the samples (pItem = 0.8) in each iteration for clustering. The Prediction Average Clustering (PAC) method was used to calculate the PAC values for different values of K, and the K value with the minimum PAC value was chosen as the best number of clusters. The internal consistency index (ICL) was used to evaluate the stability and consistency of the clustering for different values of K.

PCA was employed in the clustering analysis to assess the separation of various subtypes according to their expression patterns. The ‘survival’ and ‘survminer’ packages were used to perform overall survival (OS) and progression-free survival (PFS) analyses to assess the prognostic differences among the subtypes. The ‘estimate’ package was used to analyze the tumor microenvironment of clustered subtypes and explore the tumor immune characteristics of each subtype. The ‘enrichplot’, ‘clusterProfiler’, and ‘org.Hs.eg.db’ packages were used to conduct Gene Ontology (GO), Kyoto Encyclopedia of Genes and Genomes (KEGG), and Gene Set Enrichment Analysis (GSEA), exploring the functional enrichment status of each clustered subtype. Finally, the ‘ggalluvial’ package was employed to create Sankey diagrams, which help uncover the link between subtypes and established immune phenotypes, highlighting the connection between subtypes and the immune microenvironment.

### 2.3 Weighted gene co-expression network analysis

Use the ‘WGCNA’ package to conduct WGCNA analysis on clustered subtypes, identifying module genes closely associated with these subtypes. First, rank all genes within the clustered subtypes by standard deviation, selecting the top 25% most variable genes for further analysis. The optimal soft threshold was determined by calculating the topological fit index under different power values. Second, gene clustering was performed using the Topological Overlap Matrix (TOM), with the minimum number of module genes set to 100. Finally, genes most closely associated with the subtypes were selected based on the criteria of Module Membership (MM) > 0.8 and Gene Significance (GS) > 0.5.

### 2.4 Univariate cox regression analysis and machine learning

The module genes closely related to the clustering subtypes obtained from WGCNA were intersected with the PCD-DEGs gene set. The intersected genes underwent a univariate Cox regression analysis to assess their prognostic relevance. Subsequently, five machine learning algorithms were utilized to refine the selection of prognostic genes. The packages ‘e1071,’ ‘kernlab,’ and ‘caret’ were used for SVM-RFE. The Random Forest analysis was conducted using the ‘randomForest’ package. The ‘XGBoost’ package was used to perform XGBoost analysis. The ‘mclust’ package was employed for Gaussian Mixture Model (GMM) analysis. Finally, the results from the five machine learning algorithms were intersected to identify overlapping genes, which were recognized as core prognostic PCD-DEGs.

### 2.5 Development and validation of the risk assessment model

Using LASSO regression analysis, we identified the optimal prognostic gene combination to build a Cox proportional hazards model. We divided the population into risk groups based on risk scores and conducted prognosis-related analyses. The risk score calculation formula is: risk score = ∑(Xi * Yi), where X represents the coefficient and Y indicates the gene expression level.

We calculated the median of the risk scores and divided the population into high-risk and low-risk groups based on this median. The clustering subtypes were analyzed in conjunction with the risk groups to understand the prognostic performance of the clustering subtypes under different risk statuses. A survival status plot was created to display the survival and death conditions of patients across various risk categories. Differences in survival between these groups were validated through OS analysis. We used the ‘timeROC’ package to conduct ROC curve analysis, evaluating the model’s predictive performance for the prognosis of UCEC patients at various time points.

### 2.6 Development and verification of the nomogram

We performed univariate and multivariate Cox regression analyses on clinical features such as risk score, age, stage, grade, and body mass index (BMI), using hazard ratios and p-values to determine the prognostic significance of each clinical feature. We used the ‘rms’ and ‘regplot’ packages to construct a nomogram. We assessed the nomogram’s predictive accuracy for OS in UCEC patients using calibration curves. We plotted cumulative risk curves to explore long-term survival trends for patients at different risk levels. We constructed time-dependent ROC curves to assess the prognostic accuracy of the nomogram for UCEC patient prognosis at various time points. We built decision curves to observe the clinical net benefit of the nomogram at different time intervals.

### 2.7 Tumor mutational burden

Retrieve tumor mutation data for UCEC patients from the TCGA database using the ‘maftools’ package. Analyze the gene mutation status among different risk groups and examine the combined impact of risk score and TMB on patient survival outcomes.

### 2.8 Tumor immune dysfunction and exclusion

Access TIDE scores for UCEC patients from http://tide.dfci.harvard.edu/. Employ a chi-square test to evaluate the response to immunotherapy across various risk categories among UCEC patients. Generate ROC curves to determine the model’s accuracy in predicting the effectiveness of immunotherapy. Concurrently, utilize the ‘CIBERSORT’ R package for the analysis of immune infiltration.

### 2.9 Drug sensitivity analysis

Perform drug sensitivity analysis using the ‘oncoPredict’ package, drawing on data from the GDSC database. Conduct differential analysis of drug responses by comparing the half-maximal inhibitory concentration (IC50) values across different risk score groups, applying a p-value filtering threshold of less than 0.001 to identify potential targets for drug treatment.

### 2.10 Reverse transcription quantitative polymerase chain reaction

Isolate total RNA from samples using the Trizol reagent (RNAiso Plus, TaKaRa Biotechnology, SD1412). Convert 1000 ng of RNA into cDNA using the PrimeScript RT Reagent Kit (TaKaRa Biotechnology, RR036A). Perform amplification using SYBR Green qPCR Master Mix (TOLOBIO, Shanghai, China, #22204) according to the manufacturer’s guidelines. Amplification primers were synthesized by Beijing Tsingke Biotech Co., Ltd. (Beijing, China) (**Table 2**). Carry out real-time PCR amplification on a Gentier 96E/96R Fully Automated PCR System (Xi’an Tian Long Technology, China). Determine relative gene expression levels using the 2−ΔΔCT method, with β-actin serving as the reference gene.

**Table 2:** The primers of core prognostic PCD-DEGs and β-actin.

Gene name	Primer orientation	Sequences
ATP6V1C2	Forward	GAAGCCGCTGCCTCCC
	Reverse	ATCTGGCCATCTCCTCCCTT
SRPX	Forward	AGCTTCCCAGATACCCCGT
	Reverse	TTGTTGGGTTCTGCAATGCG
NT5E	Forward	CTCCTCTCAATCATGCCGCT
	Reverse	TGGATTCCATTGTTGCGTTCA
β-actin	Forward	CCTTCCTGGGCATGGAGTC
	Reverse	TGATCTTCATTGTGCTGGGTG

### 2.11 Statistical analysis

Conduct statistical analysis using R software (version 4.3.3), with data visualization supported by the ‘ggplot2’ and ‘ggpubr’ packages. Assess differences between two groups using the Wilcoxon rank-sum test, log-rank test, or chi-square test. Apply the Benjamini-Hochberg method to control the false discovery rate (FDR) in multiple comparisons. Consider FDR values less than 0.05 as significant for differential gene expression and gene set enrichment analyses. For analyses without multiple comparisons (e.g., survival analysis), use a significance threshold of p < 0.05. Design all analyses to ensure robustness and reproducibility of results.

## 3 Results

### 3.1 Identification of PCD-DEGs and consensus clustering subtypes

**Figure 2:**
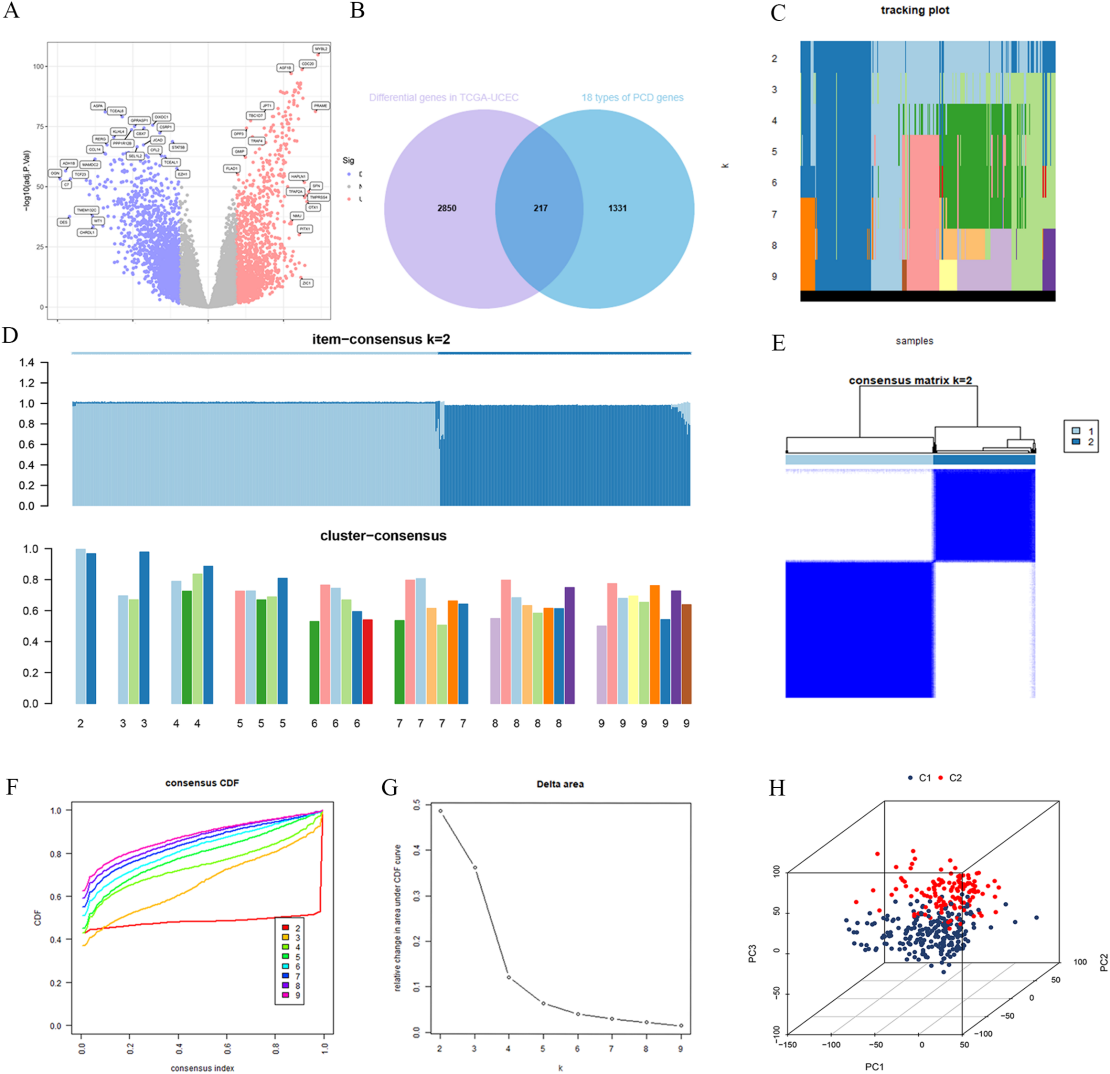
Consensus clustering was performed for subtyping after intersecting the differentially expressed genes from TCGA-UCEC with PCD-related genes. **(A)** TCGA-UCEC differential genes. **(B)** The intersection of TCGA-UCEC differential genes and PCD genes. **(C)** Tracking plot for cluster assignments across different k values. **(D)** Item-consensus and cluster-consensus plots for different k values. **(E)** Consensus matrix for k=2. **(F)** Consensus CDF curves for evaluating cluster stability across different cluster numbers (k). **(G)** Relative change in area under CDF curve vs. number of clusters (k). **(H)** 3D principal component analysis plot of clusters C1 and C2.

The TCGA-UCEC dataset was subjected to differential expression analysis, revealing 3,067 DEGs as shown in **Figure 2A**. By intersecting these DEGs with a previously compiled list of 1,548 PCD genes, we identified 217 PCD-related DEGs (PCD-DEGs) (**Figure 2B**; **Supplementary Table S3**).

To explore the underlying structure of the data, we performed consensus clustering using the expression profiles of the PCD-DEGs. As the number of clusters (K) increased from 2 to 9, the most stable sample grouping emerged at K=2 (**Figures 2C, D**), which exhibited the highest intra-group similarity and the lowest inter-group similarity (**Figures 2E**). The cumulative distribution function (CDF) curve confirmed the minimal relative area at K=2 (**Figure 2F**), indicating optimal cluster stability at this division (**Figure 2G**). Consequently, 543 UCEC patients were effectively classified into two subgroups: C1 (n=322) and C2 (n=221). PCA demonstrated a clear separation between these subgroups (**Figure 2H**), suggesting significant differences in their gene expression patterns.

### 3.2 Biological mechanisms and survival analysis of C1 and C2 subtypes

**Figure 3:**
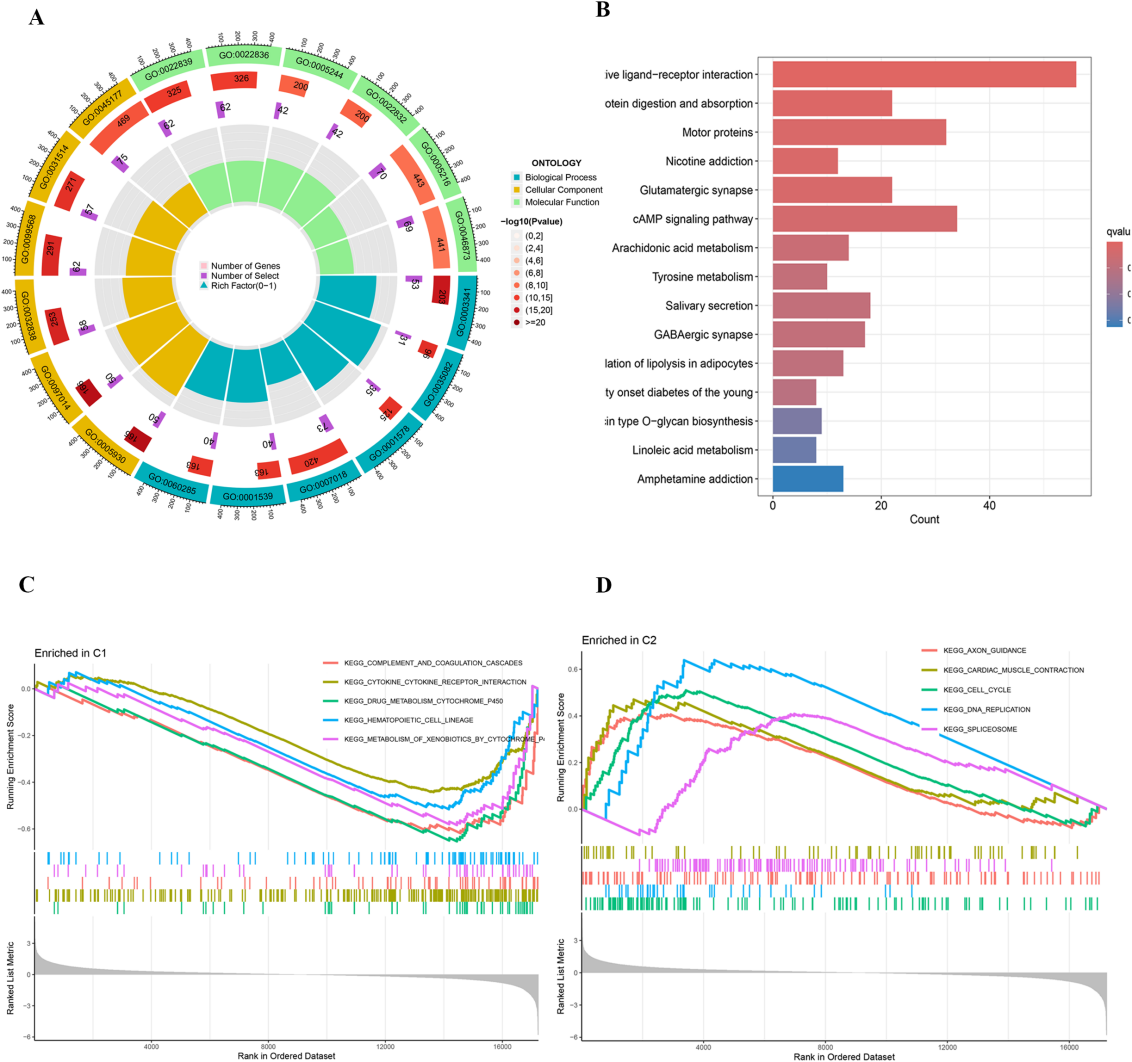
**(A)** Circular plot of GO enrichment analysis. **(B)** Bar plot of KEGG pathway enrichment analysis. **(C, D)** GSEA plots of KEGG pathway enrichment.

**Figure 4:**
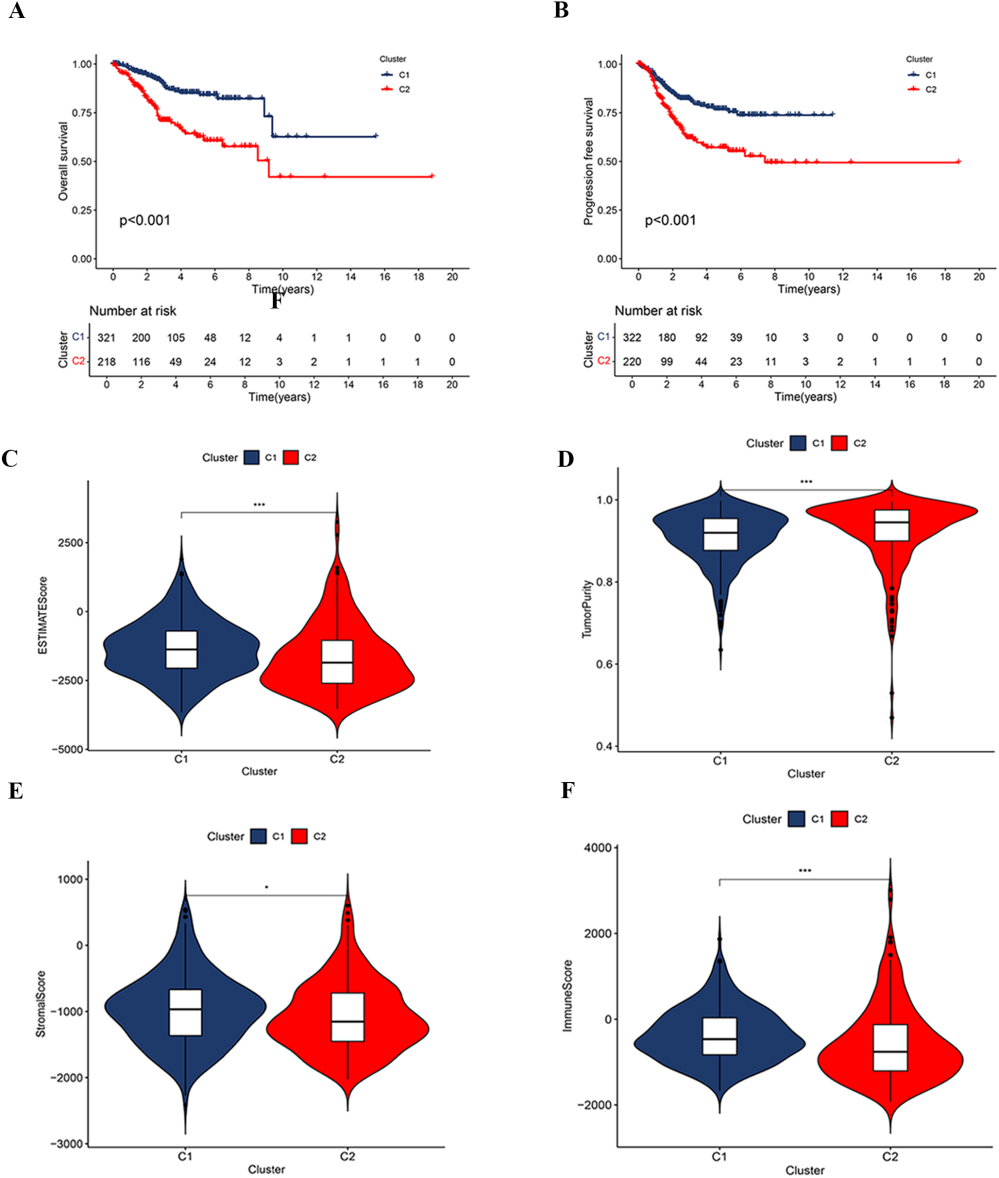
Correlation analysis based on consensus clustering subtypes. **(A, B)** Kaplan-Meier survival curves for OS and PFS between clusters C1 and C2. **(C–F)** Comparison of tumor immune microenvironment characteristics between clusters C1 and C2, including the ESTIMATE score **(C)**, tumor purity **(D)**, stromal score **(E)**, and immune score **(F)**.

Further differential expression analysis between these subtypes identified 162 genes with distinct expression profiles. GO enrichment analysis revealed significant enrichment of these genes in biological processes such as cilium movement, axoneme structure, and monoatomic ion-gated channel activity (**Figure 3A**). Moreover, KEGG pathway analysis showed significant enrichment in pathways including “Protein Digestion and Absorption”, “Motor Proteins”, and “Neuroactive Ligand–Receptor Interaction” (**Figure 3B**). GSEA indicated a strong association of the C1 subtype with immune response, metabolic functions, and hematopoietic system processes (**Figure 3C**), while the C2 subtype appeared more closely related to neural system development, myocardial function, cell proliferation, and regulation of gene expression (**Figure 3D**). Survival analysis highlighted significant differences in OS and PFS between the C1 and C2 subtypes (p < 0.001), with the C2 subtype associated with a poorer prognosis (**Figures 4A, B**).

### 3.3 Analysis of the tumor microenvironment and immune subtypes in C1 and C2 classifications

The analysis of the tumor microenvironment demonstrated that the C1 subtype exhibited higher ESTIMATE, Immune, and Stromal Scores compared to the C2 subtype, indicating lower tumor purity (**Figures 4C–F**). Further investigation into the relationship between clustering subtypes and immune subtypes revealed distinct patterns of immune subtype distribution between C1 and C2 (**Supplementary Figure S1**). Specifically, the C1 subtype was predominantly associated with a reparative immune response, while the C2 subtype exhibited characteristics of a more active immune response.

### 3.4 WGCNA

**Figure 5:**
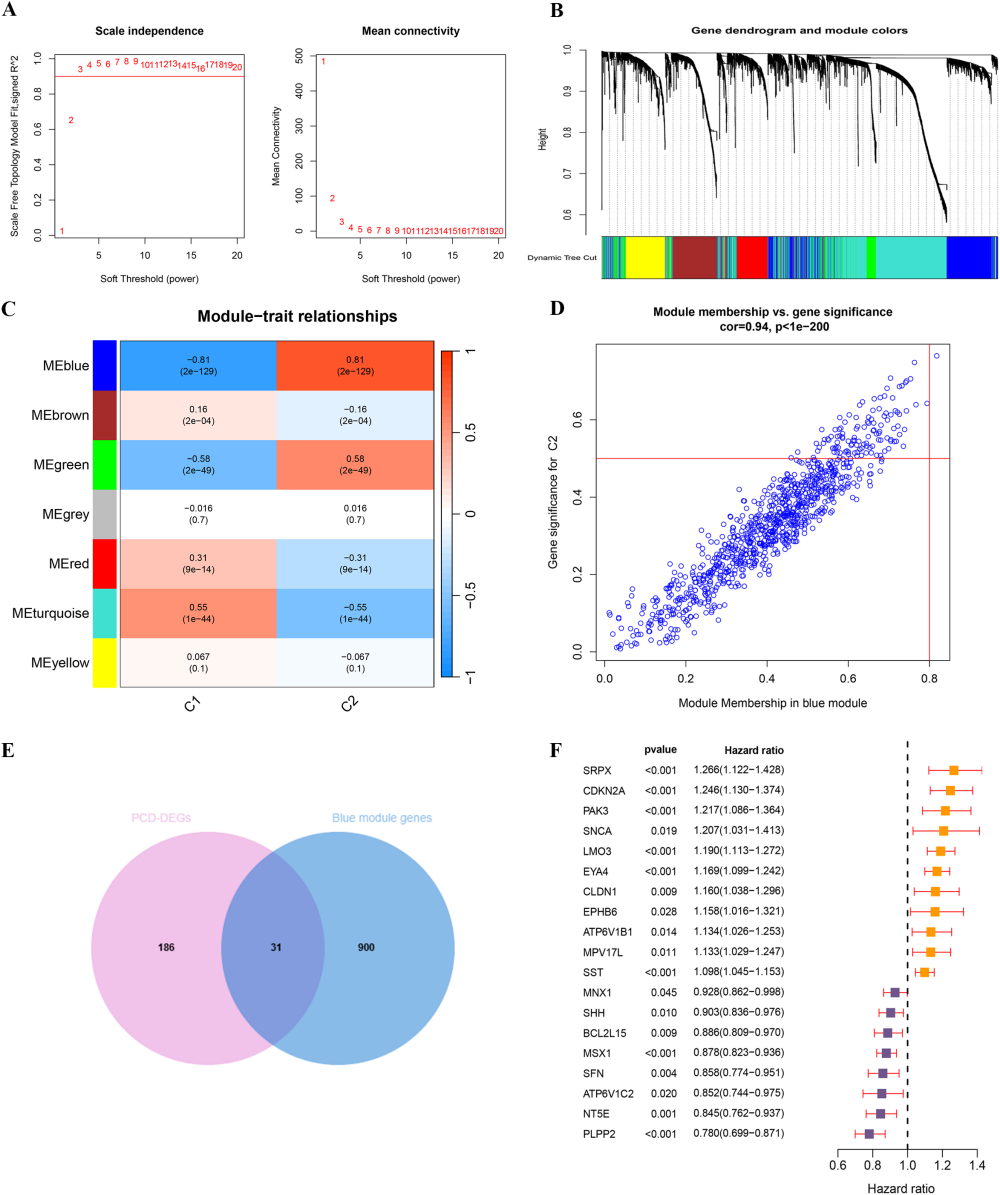
WGCNA analysis and identification of key PCD prognostic genes based on consensus clustering subtypes. **(A)** Scale independence and mean connectivity analysis for soft threshold selection. **(B)** Gene dendrogram and module color assignment. **(C)** Heatmap of module–trait relationships. **(D)** Correlation between module membership and gene significance in the blue module. **(E)** The intersection of PCD-DEGs and blue module genes. **(F)** UniCox regression analysis of 19 PCD prognosis-related genes.

We conducted a WGCNA using TCGA-UCEC expression data, focusing on the correlation with subtypes C1 and C2. To establish a scale-free network, a soft threshold power b=3 (R²=0.90) was selected (**Figure 5A**). This analysis successfully identified seven distinct gene modules (**Figure 5C**). The classification of these modules and the patterns of gene co-expression were visualized through a dendrogram and a heatmap (**Figure 5B**; **Supplementary Figure S2**). Notably, 931 genes within the blue module (**Supplementary Table S4**) demonstrated significant correlations—both negative and positive—with the C1 and C2 subtypes, respectively. This suggests that the blue gene module plays a pivotal role in the characteristics of the C2 subtype. A correlation coefficient of 0.94 between MM and GS for the blue gene module underscores a strong positive association (**Figure 5D**), warranting further detailed investigation of this module.

### 3.5 Identification of core prognostic PCD-DEGs

Through the intersection of 217 PCD-DEGs with the previously identified blue gene module via WGCNA, we identified 31 overlapping genes (**Figure 5E**; **Supplementary Table S5**). Univariate Cox regression analysis of these genes revealed that 19 PCD-DEGs were significantly associated with clinical prognosis (**Figure 5F**).

**Figure 6:**
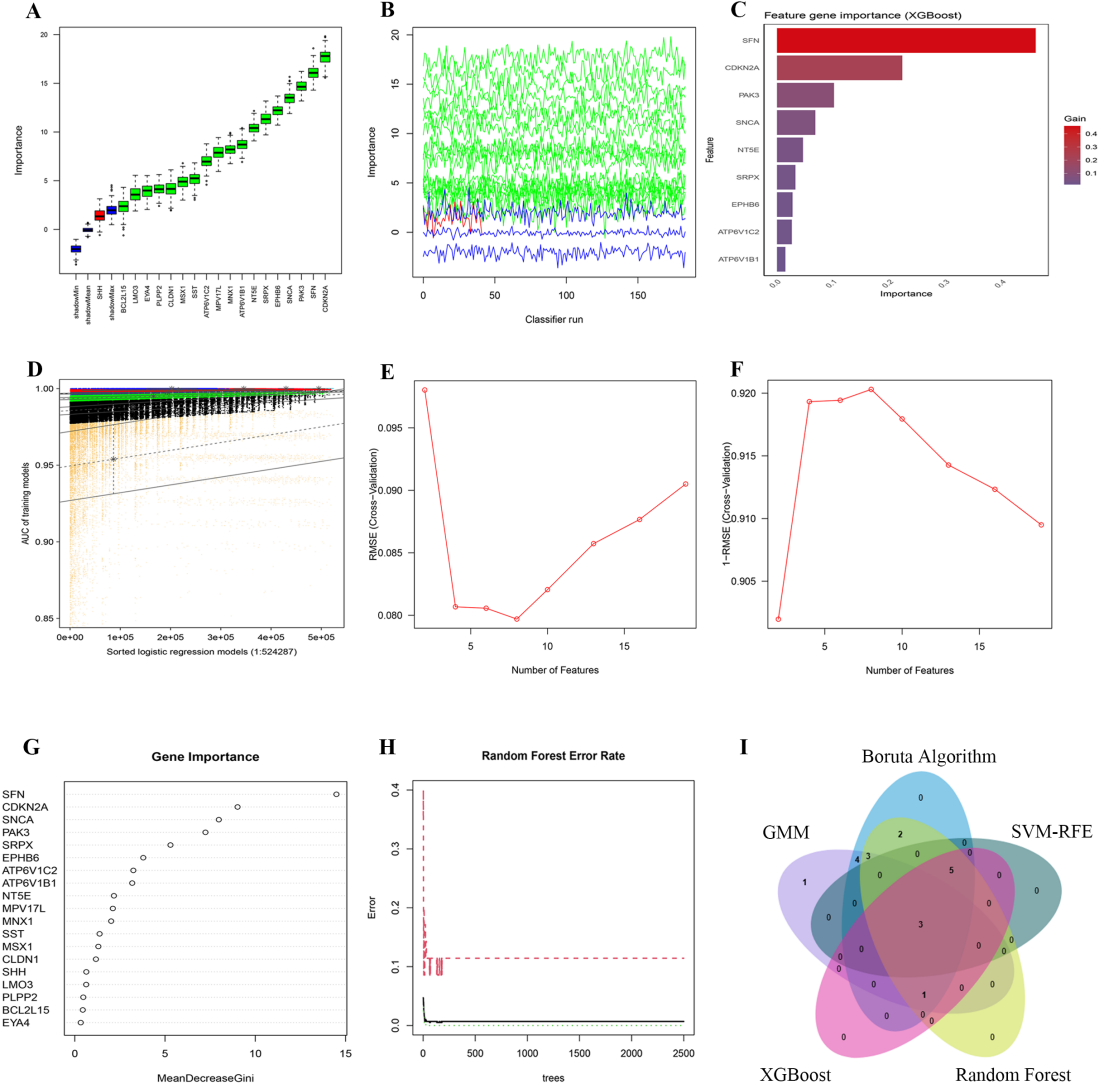
Dimensionality reduction and feature selection for 19 PCD-related prognosis genes using machine learning algorithms. **(A, B)** Feature selection with the Boruta algorithm: **(A)** shows importance scores and **(B)** displays importance trends across classifier runs. **(C)** Feature ranking with the XGBoost algorithm, highlighting the most significant genes by importance scores. **(D)** Feature selection with the GMM algorithm, showing AUC distributions for logistic regression models. **(E, F)** Feature selection with the SVM-RFE algorithm: **(E)** shows RMSE across feature numbers and **(F)** uses 1-RMSE to indicate performance. **(G, H)** Feature selection with the Random Forest algorithm: **(G)** shows MeanDecreaseGini values and **(H)** plots error rates across different tree numbers. **(I)** The intersection of five machine learning algorithms identified 3 Hub-DEGs.

To discern key prognostic genes among these 19 PCD-DEGs, we utilized five sophisticated machine learning algorithms, each chosen for its unique capabilities in feature selection and dimensionality reduction to ensure the reliability and robustness of our findings. The Boruta algorithm, which extends the Random Forest approach, was employed to ascertain the relevance of each feature by comparing it to randomly generated shadow features. After 1000 iterations, Boruta highlighted 18 genes of significant importance (**Figures 6A, B**). This method is particularly valuable in handling high-dimensional datasets by eliminating superfluous or irrelevant variables and preserving the stability of essential features.

Additionally, we applied the XGBoost algorithm, a gradient-boosting decision tree framework, to evaluate the importance of features using the Gain metric. Genes with a Gain value above 0.01 were deemed significant. This analysis pinpointed 9 crucial genes (**Figure 6C**). Owing to its efficiency in detecting nonlinear relationships and complex interactions, XGBoost is exceptionally suited for the analysis of gene expression data.

To investigate latent patterns and interactions among multiple genes, GMMs were employed. We generated a comprehensive set of 524,287 model combinations, selecting those with area under the curve (AUC) values approaching unity. This method successfully pinpointed 12 genes of significant importance (**Figure 6D**). As an unsupervised probabilistic model, GMMs are particularly adept at uncovering hidden structures within data, making them invaluable for modeling interactions between genes.

The SVM-RFE algorithm was applied to refine feature selection by systematically excluding features that made minimal contributions to classification accuracy. Following cross-validation, SVM-RFE effectively minimized classification errors and delineated eight critical genes as optimal features for classification (**Figures 6E, F**). This technique efficiently eliminates redundant features while preserving those of utmost relevance.

Subsequently, the Random Forest algorithm was utilized to assess feature importance based on Mean Decrease Gini scores. Only genes with importance scores exceeding 1.0 were retained, identifying 15 genes of significant importance (**Figures 6G, H**). Known for its robustness against noise and its capability to manage high-dimensional datasets, Random Forest proved to be an effective tool for feature selection within this study.

By synthesizing the outcomes derived from all employed algorithms, three genes—NT5E, SRPX, and ATP6V1C2—were consistently identified as significant across all methodologies. These genes were thus recognized as core prognostic PCD-DEGs (**Figure 6I**; **Supplementary Table S6**). The adoption of a multi-algorithm strategy not only enhances the reliability of the gene selection but also minimizes potential biases inherent in individual methods. This approach leverages the complementary strengths of each algorithm, yielding results that are both robust and interpretable.

### 3.6 Evaluation of the prognostic risk score model’s performance

**Figure 7:**
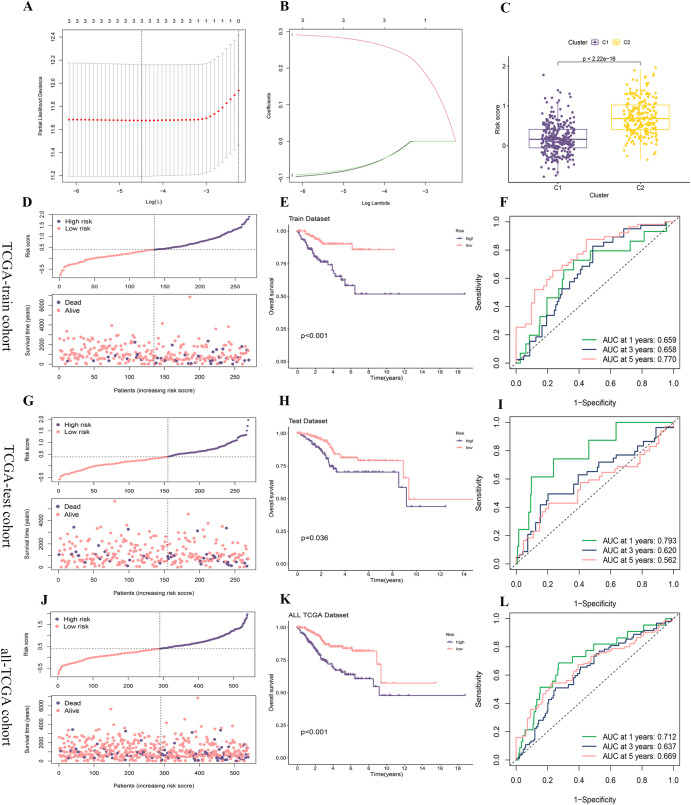
Construction and validation of the risk score model. **(A, B)** A prognostic model was constructed in the TCGA-train cohort using LASSO-COX regression analysis, with **(A)** showing the selection of the optimal lambda and **(B)** displaying the coefficient profiles as a function of log (lambda). **(C)** Risk score comparison between clusters C1 and C2. **(D, G, J)** The distribution of risk scores and survival status of each patient in the TCGA-train, TCGA-test, and all-TCGA cohorts. **(E, H, K)** Kaplan-Meier survival analysis for high- and low-risk groups in the TCGA-train, TCGA-test, and all-TCGA cohorts. **(F, I, L)** Time-dependent ROC curves for predicting 1-, 3-, and 5-year survival in the TCGA-train, TCGA-test, and all-TCGA cohorts.

The TCGA-train cohort consisted of 270 patients, while the TCGA-test cohort included 269 patients, following a random division in a 1:1 ratio. Utilizing three core prognostic PCD-DEGs (NT5E, SRPX, and ATP6V1C2) identified earlier, a risk score model was constructed based on the TCGA-train cohort. The model demonstrated optimal predictive performance at λ = 3, identifying ATP6V1C2, SRPX, and NT5E as the most effective gene combination for constructing the model (**Figure 7A**). The regression coefficients for ATP6V1C2, SRPX, and NT5E were -0.06814, 0.27367, and -0.06628, respectively (**Figure 7B**). An integrated analysis that combined clustering subtypes with risk scores revealed that the risk score associated with the C2 cluster was significantly higher than that for the C1 cluster (**Figure 7C**).

The distribution of risk scores and survival status plots indicated that the model effectively differentiates between patients with varying prognostic risks, with the high-risk group displaying a higher mortality rate (**Figures 7D, G, J**). In the OS analysis, the survival rate of the high-risk group decreased more rapidly over time, suggesting poorer survival outcomes (**Figures 7E, H, K**). Additionally, the analysis of the ROC curve confirmed that the model proficiently predicts both short-term and long-term survival outcomes for patients with UCEC, although it exhibits slightly weaker performance in predicting mid-term survival outcomes (**Figures 7F, I, L**).

### 3.7 Examination of the nomogram model’s capabilities

**Figure 8:**
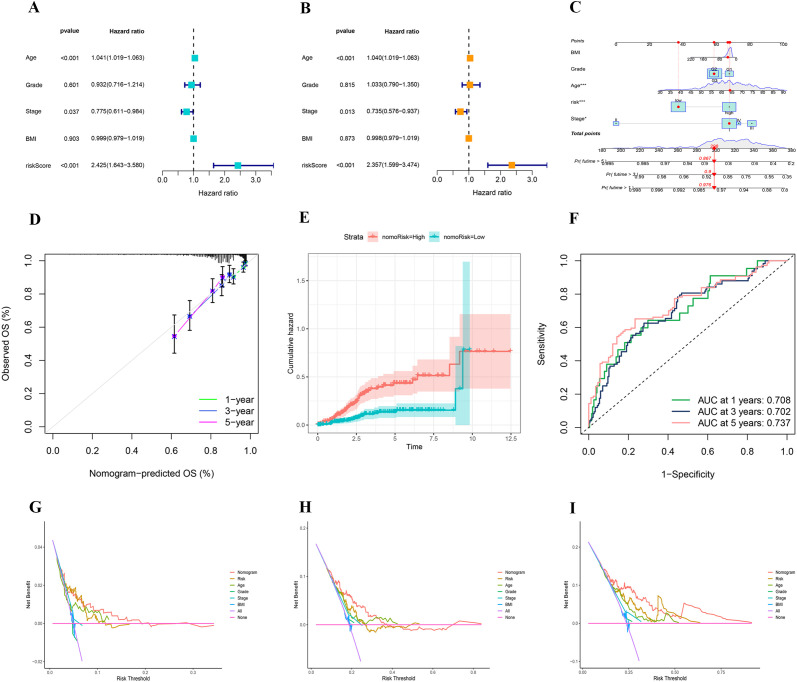
**(A, B)** Univariate and multivariate analyses showing the prognostic value of clinical variables and risk score. **(C)** A nomogram integrating clinical features and risk score to predict 1-, 3-, and 5-year overall survival probabilities. **(D)** Calibration plot assessing the accuracy of the nomogram in predicting 1-, 3-, and 5-year overall survival. **(E)** Comparison of cumulative hazard between high- and low-risk groups defined by nomogram-derived risk stratification. **(F)** Time-dependent ROC curves showing the predictive performance of the nomogram for 1-, 3-, and 5-year overall survival. **(G–I)** Decision curve analysis for 1-, 3-, and 5-year survival.

Following the Cox regression analysis, risk score, stage, and age were identified as independent predictors of patient prognosis (**Figures 8A, B**). These factors were subsequently utilized to construct a nomogram. To enhance the clinical utility of the nomogram, BMI and tumor grade were also included in the model (**Figure 8C**). Calibration curves demonstrated that the predicted values from the nomogram closely aligned with the actual observed values at each time point, affirming the model’s accuracy in forecasting survival rates at 1, 3, and 5 years (**Figure 8D**). The cumulative risk curve illustrated that the high-risk group exhibited elevated cumulative risk values, accumulating more rapidly over time (**Figure 8E**). The ROC curve analysis displayed AUC values for the nomogram model of 0.708 at 1 year, 0.702 at 3 years, and 0.737 at 5 years, highlighting the model’s strong discriminative ability (**Figure 8F**). The DCA indicated that the nomogram effectively integrates multiple variables, offering superior predictive power and clinical utility compared to models based on a single variable. The nomogram achieves the optimal clinical net benefit within the moderate-risk threshold range (**Figures 8G–I**).

### 3.8 The association between TMB, risk score, and patient outcomes

**Figure 9:**
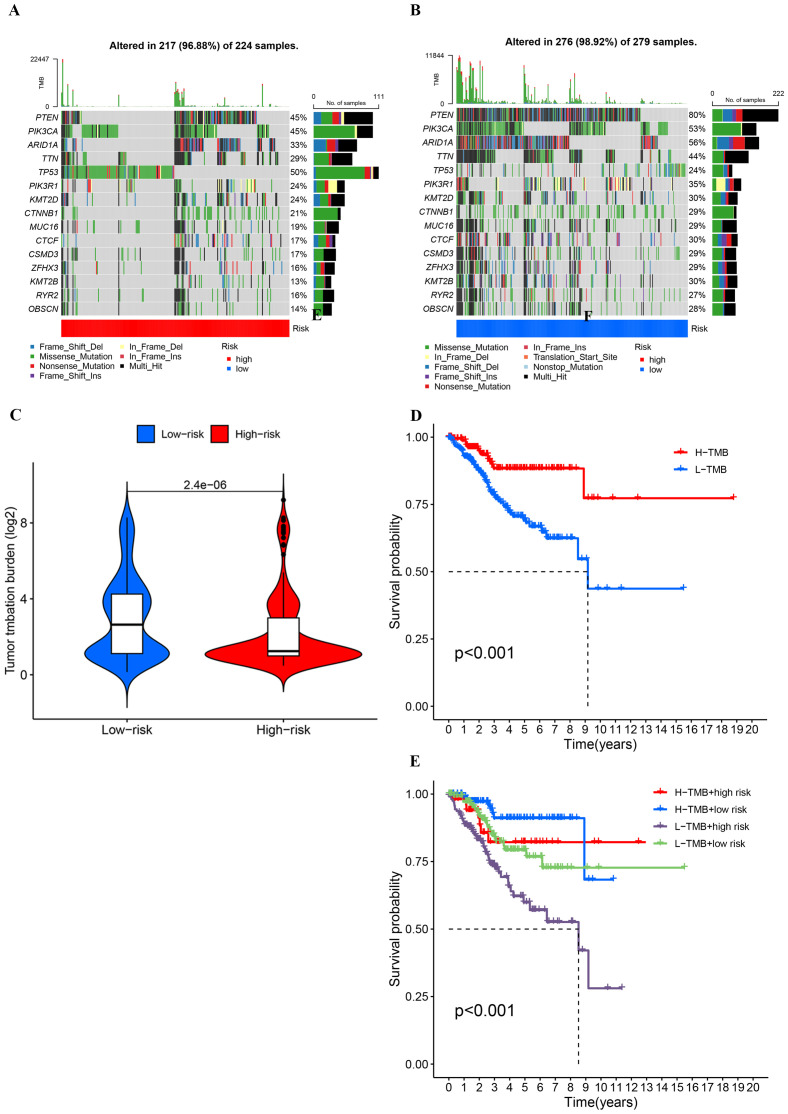
**(A, B)** Comparison of mutation frequencies and types between high- and low-risk groups based on risk scores. **(C)** Comparison of tumor mutationburden (log2-transformed) between low-risk and high-risk groups.**(D, E)** Kaplan-Meier survival curves stratified by tumor mutation burde and risk groups.

The waterfall plot depicting gene mutation characteristics effectively illustrates the distribution and frequency of mutations within the high- and low-risk groups. Predominantly, missense mutations are observed in both groups. Within the high-risk group, TP53, PTEN, and PIK3CA are identified as the genes with the highest mutation frequencies, which likely act as principal drivers of tumor progression and deterioration (**Figure 9A**). Conversely, in the low-risk group, PIK3CA, PTEN, and ARID1A exhibit the highest mutation frequencies, suggesting a correlation with the initial stages of tumor development (**Figure 9B**). Notably, patients classified as high-risk demonstrate lower TMB levels (**Figure 9C**) and significantly reduced survival rates (**Figure 9D**). A detailed subgroup analysis was conducted to further delineate the relationship among TMB levels, risk scores, and patient prognosis. This analysis indicated that patients with elevated TMB levels coupled with low-risk scores manifest the highest survival rates, whereas those with reduced TMB levels and high-risk scores experience the lowest survival rates (**Figure 9E**). These findings underscore the critical role of TMB and risk scores in prognostic assessments and support their potential utility as clinical prognostic biomarkers.

### 3.9 Immune evasion and immunoinfiltration

**Figure 10:**
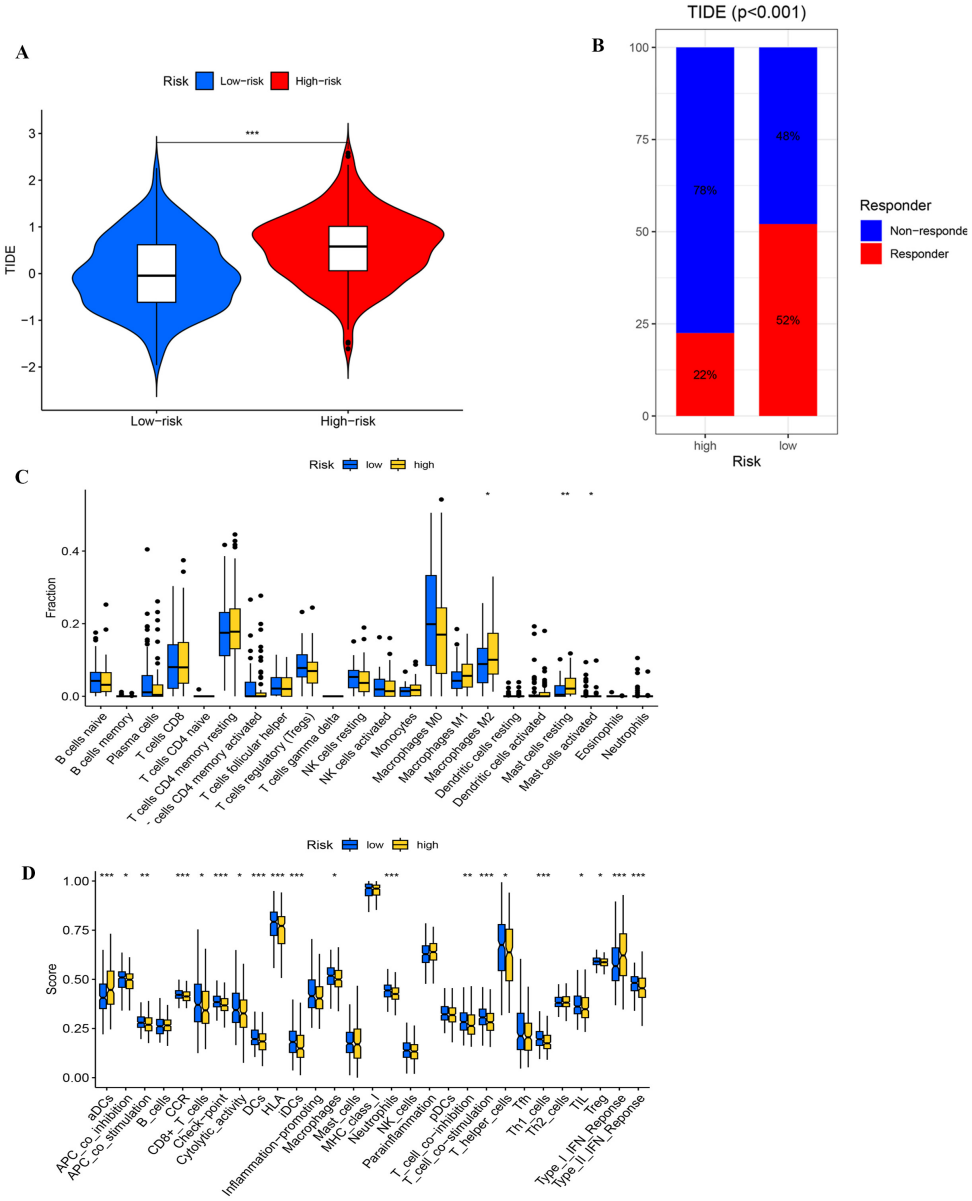
**(A)** Comparing TlDE scores between high- and low-risk groups. **(B)** The proportion of responders and non-responders in high- and low-risk groups based on TIDE scores. **(C)** Differential analysis of immune cells. **(D)** Functional analysis of immune cells. *, ** , and *** , means *p*-values less than 0.05, 0.01, and 0.001, respectively.

The cohort classified as high-risk is characterized by an increased TIDE score, indicative of an enhanced ability for immune evasion (**Figure 10A**). Analysis of immunotherapy responses shows that responders have significantly lower TIDE scores compared to non-responders (p < 0.001). Within the low-risk group, it is projected that 52% might benefit from immunotherapy, in stark contrast to only 22% in the high-risk group (**Figure 10B**). This disparity suggests a diminished immunotherapeutic response in the high-risk group. The ROC curve analysis confirms that the model is capable of effectively predicting immunotherapy responses and immune evasion in UCEC patients (**Supplementary Figure S3**).

In the immune cell differential analysis (**Figure 10C**), macrophages M2 are significantly more prevalent in the high-risk group, potentially facilitating tumor growth by secreting immunosuppressive cytokines, such as IL-10 and TGF-β, which inhibit T cell activity. Moreover, both resting and activated mast cells are found in greater abundance in the high-risk group, with activated mast cells possibly altering the tumor microenvironment and promoting immune evasion through the release of pro-inflammatory factors. In the immune-related functional analysis (**Figure 10D**), the high-risk group exhibits elevated scores for activated dendritic cells and Type I interferon response, suggesting increased inflammation and antiviral immunity, potentially accompanied by immune dysregulation. Conversely, the low-risk group displays elevated scores for HLA-related gene expression and T cell co-stimulation, indicating superior antigen-presentation capability and T cell activation, which contribute to a more robust anti-tumor immune response. These observations highlight a trend towards immunosuppression and inflammation in the high-risk group’s immune microenvironment, whereas the low-risk group demonstrates more effective anti-tumor immune characteristics.

### 3.10 Screening potential anticancer drugs

**Figure 11:**
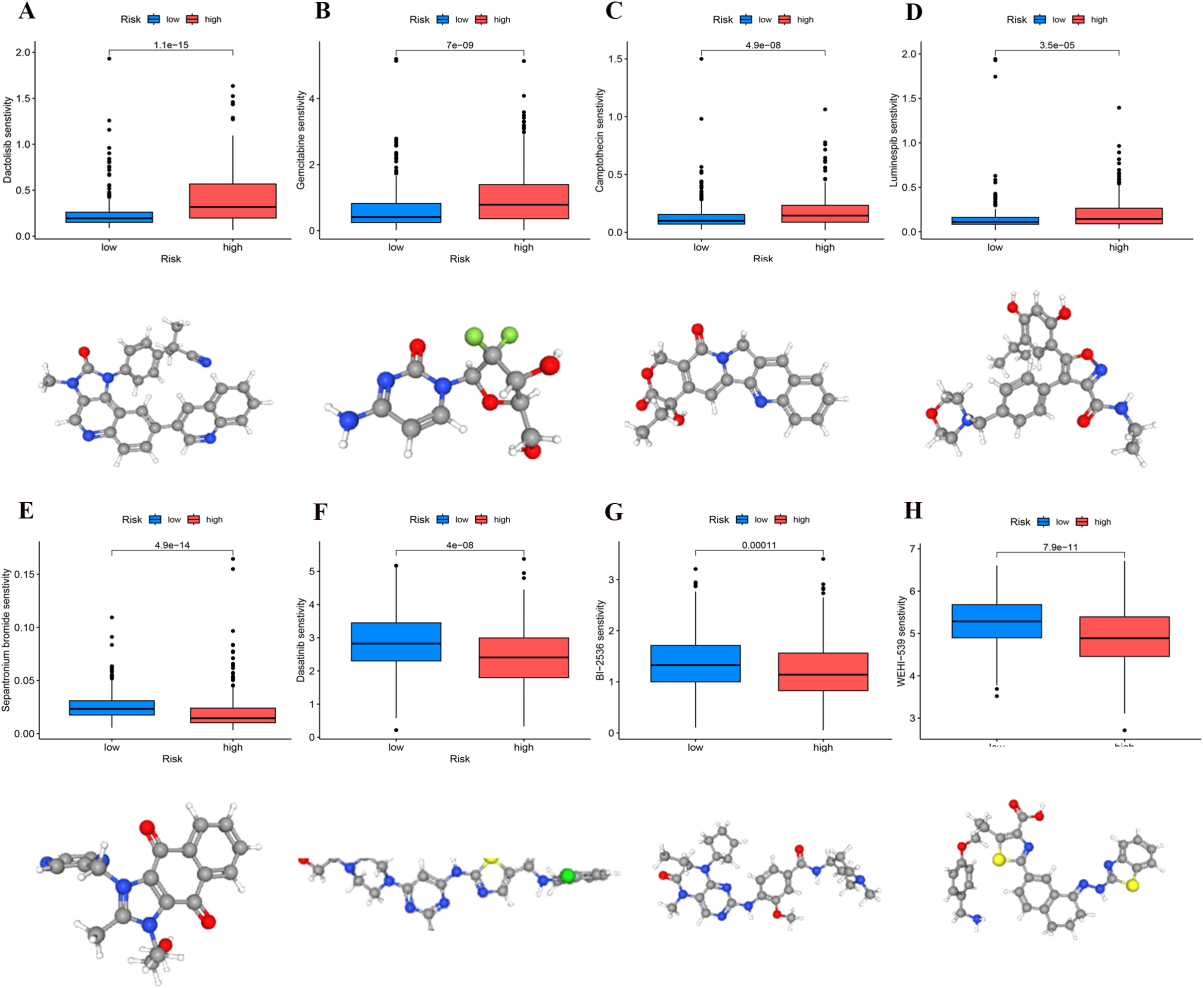
Identification of sensitivity drugs for high- and low-risk groups through differential analysis of IC50. **(A)** Dactolisib. **(B)** Gemcitabine. **(C)** Camptothecin. **(D)** Luminespib. **(E)** Sepantronium bromide. **(F)** Dasatinib. **(G)** BI-2536. **(H)** WEHI-539.

We conducted a comparative analysis of IC50 concentrations for various anticancer agents among patient cohorts stratified by risk levels. The findings indicated that the IC50 values for Dactolisib, Luminespib, Camptothecin, and Gemcitabine were significantly lower in the high-risk group (p < 0.001), suggesting enhanced drug sensitivity in patients with elevated risk scores (**Figures 11A–D**). In contrast, the IC50 values for WEHI-539, Dasatinib, BI-2536, and Sepantronium bromide demonstrated significant reductions in the low-risk group (p < 0.001), indicating higher efficacy in these patients (**Figures 11E–H**). These observations suggest that patients classified within high- and low-risk categories of UCEC might realize substantial clinical benefits from these targeted therapies.

### 3.11 Expression validation of core prognostic PCD-DEGs

**Figure 12:**
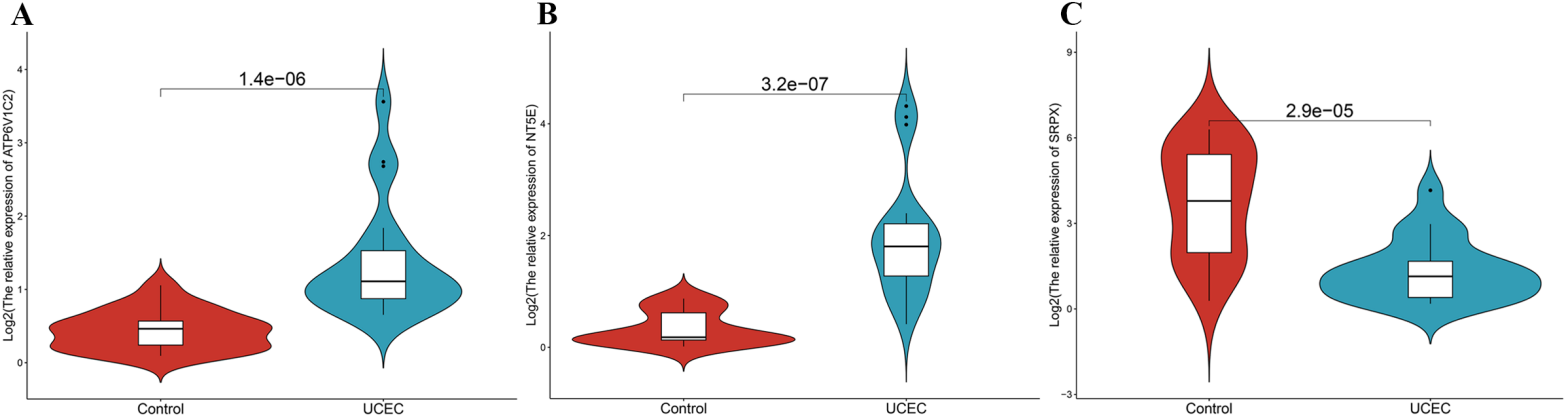
The mRNA expression validation between control and UCEC tissues by RT-qPCR. **(A)** ATP6V1C2. **(B)** NT5E. **(C)** SRPX.

To substantiate the mRNA expression of pivotal genes, biopsy samples were collected from 20 UCEC patients and 20 control subjects. RT-qPCR analysis revealed that the mRNA levels of SRPX were decreased in the UCEC samples relative to the controls, whereas the levels of ATP6V1C2 and NT5E were elevated (**Figures 12A–C**). Further validation of these expression trends within our model involved examining GEO datasets pertinent to endometrial cancer (e.g., GSE17025). The analysis confirmed significant differential expression of SRPX and NT5E between normal and cancerous tissues, although ATP6V1C2 did not exhibit notable differences (**Supplementary Figure S4**).

## 4 Discussion

Recent research has suggested that PCD is critical in developing, advancing, and managing UCEC. To be specific, some studies have reported that the dysregulated expression of apoptosis-related genes, including BAX and BCL2, is significantly linked to immune evasion and aberrant proliferation in UCEC (15, 16). The excessive expression of the ferroptosis regulator GPX4 is crucial in the advancement of UCEC and resistance to chemotherapy (17, 18). The unusual expression of PCD-associated genes significantly affects the apoptosis, proliferation, and drug resistance properties of tumor cells. Thus, incorporating PCD-related genes into clinical prognostic models for UCEC provides insights into the tumor’s biological mechanisms and plays a crucial role in identifying novel therapeutic targets.

UCEC patients were classified into two cluster subtypes, C1 and C2, in accordance with the expression profile of PCD-DEGs. The C2 subtype exhibited higher tumor purity and immune suppression, suggesting that genes associated with the C2 subtype may be associated with an immunosuppressive microenvironment. Moreover, tumor cells in the C2 subtype may possess greater invasiveness. Using WGCNA, the module genes most strongly associated with the C2 subtype were identified for subsequent analyses. This approach helps elucidate the immunosuppressive mechanisms and poor prognostic characteristics of the C2 subtype. Furthermore, it is crucial in optimizing clinical prediction models and identifying novel therapeutic targets.

As high-throughput sequencing technologies continue to develop, the generation of large-scale biological data has provided abundant resources for cancer research. Notably, traditional bioinformatics methods have certain limitations when dealing with complex multidimensional data. Machine learning (ML) techniques exhibit exceptional capabilities in pattern recognition and dimensionality reduction. Thus, these techniques can offer new pathways for uncovering cancer biology characteristics. ML has shown significant potential, particularly in elucidating tumor molecular mechanisms, identifying prognostic biomarkers, and enabling personalized treatment strategies (19, 20). In recent years, ML techniques have been widely applied in breast cancer, lung cancer, and gastric cancer for gene feature selection and multi-omics data integration, effectively improving the accuracy of disease prediction models (21–23). Therefore, applying ML to the screening and dimensionality reduction of endometrial cancer-related genes can enhance the efficiency of core gene identification. Furthermore, it can also provide important molecular targets for clinical practice.

This study utilized five advanced machine learning techniques that were employed to screen and reduce the dimensionality of PCD-related prognostic genes, aiming to identify core prognostic genes. Among these methods, the Boruta Algorithm is a feature selection method that relies on the random forest algorithm. It identifies features that significantly affect model performance by evaluating gene importance scores (24). The XGBoost algorithm is an efficient gradient-boosting decision tree method. It excels at handling nonlinear data and complex feature interactions. Thus, it is suitable for high-dimensional datasets (25). The Random Forest algorithm constructs multiple decision trees and uses a voting mechanism, providing robust feature selection capabilities (26). The SVM-RFE algorithm is an iterative feature reduction method that effectively removes redundant genes, improving model simplicity (27). The GMM algorithm is an unsupervised probabilistic model that identifies key features by uncovering latent distribution patterns within the data (28). Through cross-validation using these five algorithms, three core prognostic PCD-DEGs—SRPX, NT5E, and ATP6V1C2—were ultimately identified.

SRPX, also referred to as SRPX1 (29), ETX1 (30), and DRS (31), functions as a tumor suppressor gene and was initially recognized for its role as an inhibitor of v-src transformation. SRPX interacts with the apoptosis-inducing protein ASY/Nogo-B/RTN-xS in the endoplasmic reticulum, promoting apoptosis through endoplasmic reticulum (ER) stress-mediated signaling pathways. This process includes the sequential activation of caspase-12, caspase-9, and caspase-3 (31). Previous research has established that SRPX expression is significantly downregulated in various cancers, including those of the lung, prostate, colon, and ovary, likely due to epigenetic modifications such as DNA methylation, or the activation of oncogenes like ras and src (32–34). In cases of UCEC, dysregulated apoptosis is a characteristic feature, with tumor cells circumventing ER stress and suppressing pro-apoptotic signals to gain survival advantages. ER stress markers, including GRP78 and CHOP, are aberrantly expressed in UCEC, further impairing apoptotic signaling and facilitating tumor progression (35–37). Given the pivotal role of SRPX in regulating ER stress, its downregulation in UCEC likely plays a significant role in inhibiting these pro-apoptotic pathways, thereby contributing to tumor development and progression. Future research should aim to elucidate the molecular mechanisms underlying SRPX’s function in UCEC, particularly its interactions with ER stress markers, and assess its potential as a diagnostic biomarker and therapeutic target.

ATP6V1C2, a critical subunit of the V-ATPase complex, has increasingly been recognized for its potential role in tumorigenesis and progression. The V-ATPase complex is pivotal in regulating both intracellular and extracellular pH balance by facilitating transmembrane proton transport, thereby promoting the acidification of the TME. This acidification enhances tumor cell invasiveness and increases their metastatic potential (38). In colorectal adenocarcinoma, elevated expression of ATP6V1C2 correlates with a poor prognosis, potentially via the activation of the Wnt signaling pathway and the facilitation of epithelial-mesenchymal transition (39). Similarly, in ovarian cancer, ATP6V1B1 has been implicated in modulating tumor progression and chemotherapy resistance through the mTOR/autophagy pathway (40). In esophageal squamous cell carcinoma, an increased ATP6V1C1 to ATP6V1C2 ratio, reflecting the upregulation of ATP6V1C1 and downregulation of ATP6V1C2, has been identified as a distinctive molecular characteristic of the disease (41). Notably, the C2a isoform of ATP6V1C2 possesses unique protein-binding sites that may regulate V-ATPase activity via post-translational modifications and interactions with the cytoskeleton and signaling pathways (41). Moreover, other V-ATPase subunits, such as ATP6V0C, have been found to influence tumor invasiveness by modulating the activation of matrix metalloproteinases (42). Current studies suggest that ATP6V1C2 may regulate the functions of the V-ATPase complex and indirectly participate in critical biological processes such as TME acidification, the Wnt signaling pathway, and the mTOR/autophagy pathway. These pathways are vital in influencing tumor cell proliferation, invasion, and resistance to drugs. However, direct evidence linking ATP6V1C2 to these effects in UCEC has not yet been established, necessitating further investigative and experimental validation of its biological functions and molecular mechanisms.

NT5E, commonly known as CD73, primarily mediates the regulation of the adenosine signaling pathway through the conversion of AMP into adenosine (43). In most solid tumors, CD73 catalyzes this conversion, activating A2A and A2B receptors, which suppress the functions of T cells and NK cells, thereby facilitating immune evasion (44, 45). Contrarily, in endometrial cancer, an absence of CD73 is closely associated with increased tumor invasiveness and a poorer prognosis (46). CD73 helps anchor β-catenin to the cell membrane via the A1 adenosine receptor signaling pathway, limiting its nuclear translocation and transcriptional activity, thus inhibiting tumor-related gene expression. Conversely, the loss of CD73 leads to β-catenin nuclear translocation, activation of TCF/LEF transcription factors, and enhanced tumor progression (47). The absence of CD73 also induces alterations in the expression of zinc finger proteins and non-coding RNAs, exacerbating tumor invasiveness (47). Furthermore, CD73 maintains epithelial cell adhesion integrity through interactions with TGF-β1; however, its loss transforms TGF-β1 from a tumor suppressor into a tumor promoter, augmenting tumor cell proliferation and migration (46). Clinically, CD73 is considered a potential therapeutic target across various cancers, with the combination of CD73 inhibitors and immune checkpoint inhibitors significantly boosting antitumor efficacy (48, 49). Although CD73 has been validated as an effective prognostic biomarker in numerous cancers, its specific role in endometrial cancer remains to be elucidated, particularly its molecular mechanisms in tumor immune regulation and metastasis, and its potential for personalized therapeutic approaches.

This study represents the first to develop a comprehensive multi-gene prognostic model utilizing PCD-related genes—SRPX, ATP6V1C2, and NT5E. This model significantly enhances our understanding of the molecular mechanisms implicated in UCEC. The prognostic model, based on these genes, exhibits superior predictive accuracy and clinical utility, as evidenced by the ROC curve and survival analyses of high- and low-risk groups. The integration of the model’s risk score with clustering subtypes underscores its robust stratification capability, highlighting the pivotal roles of SRPX, ATP6V1C2, and NT5E in the molecular subtyping and prognostic forecasting of UCEC. Furthermore, the addition of the risk score to clinical variables in constructing the nomogram has enhanced the model’s interpretability and applicability in clinical settings.

The analysis of TMB and immune escape indicates that individuals in the high-risk category exhibit a reduced frequency of gene mutations, fewer types of mutations, lower TMB levels, and elevated TIDE scores, which correlate with less effective immune treatment responses. These characteristics suggest that the tumor biology of the high-risk group is relatively homogeneous, lacking diversity and adaptability. We hypothesize that low TMB may lead to insufficient antigen stimulation by the immune system, which in turn gives the tumor an advantage in immune evasion and growth, leading to a reduced response to immune therapy and ultimately affecting patient survival, consistent with existing studies (50, 51). Besides, the increased TIDE score further supports this notion, suggesting a stronger immunosuppressive microenvironment in the high-risk group. The above-mentioned findings emphasize the importance of the risk score model as a predictive indicator for immune therapy response, aiding in the identification of patients who may benefit from immunotherapy. By optimizing treatment strategies, the risk score provides scientific evidence for regulating the tumor immune microenvironment. Moreover, it guides clinical practice to improve patient treatment outcomes. In conclusion, our research provides fresh perspectives on how tumor mutation traits relate to immune therapy responses, laying a foundation for future personalized treatment strategies. Immune infiltration analysis indicates that the immune microenvironment in the high-risk group tends toward immunosuppression and an inflammatory state, while the low-risk group exhibits more effective anti-tumor immune characteristics. Subsequent research should investigate these findings through single-cell sequencing and functional experiments.

We performed a drug sensitivity analysis to improve the model’s applicability in clinical settings. Drugs selected from the high-risk group, including Dactolisib, Gemcitabine, Camptothecin, and Luminespib, exhibited strong inhibitory effects on tumor cells. Dactolisib, a PI3K/mTOR inhibitor, significantly suppressed tumor cell proliferation (52). Gemcitabine, a broad-spectrum chemotherapy agent, inhibits DNA synthesis and effectively kills rapidly proliferating tumor cells (53). Camptothecin inhibits topoisomerase I, directly causing DNA damage, and demonstrates potent anti-tumor activity (54). Luminespib inhibits HSP90 function, thereby affecting the stability of various tumor-related proteins and exhibiting strong anti-tumor activity (55). Drugs selected from the low-risk group, such as Dasatinib, Sepantronium bromide, BI-2536, and WEHI-539, are considered relatively mild. Compared to broad-spectrum chemotherapy drugs, these agents generally exhibit lower toxicity and side effects, and demonstrate good tolerance in clinical applications (56–59). For instance, Dasatinib primarily targets specific tumor markers, with relatively mild side effects, making it suitable for patients with a lower tumor burden (57). These characteristics render them a more appropriate treatment option for the low-risk patient cohort. The analysis of drug sensitivity revealed notable variations in the responses of high-risk and low-risk groups to chemotherapy agents. This finding not only validates the clinical applicability of the ATP6V1C2, SRPX, and NT5E gene model but also reinforces its importance as a theoretical foundation for advancing precision medicine. Developing personalized chemotherapy regimens for high-risk and low-risk patients, combined with targeted or immune therapies, may further enhance treatment efficacy and improve patient prognosis.

This study established a prognostic model for UCEC based on ATP6V1C2, SRPX, and NT5E. It exhibited robust predictive performance and potential for clinical implementation. The expression levels of these core genes can be determined using RT-qPCR or RNA-seq technology, which facilitates the calculation of risk scores to categorize patients into high- or low-risk groups. Patients classified as high-risk are recommended for chemotherapy (e.g., Dactolisib, Gemcitabine) or combined targeted therapy, whereas low-risk patients may benefit from targeted drugs with fewer side effects (e.g., Dasatinib or BI-2536) to optimize therapeutic outcomes. RT-qPCR is advantageous due to its low cost and rapid processing, making it suitable for clinical applications, whereas RNA-seq offers a comprehensive molecular profile, beneficial for complex cases. Despite its clinical utility, the model faces challenges in standardizing detection methods, ensuring sample quality, conducting multicenter validations, and managing costs. To address these challenges, it is imperative to establish standardized protocols, optimize sample processing, and perform multicenter validations to enhance the model’s clinical applicability. Moreover, patient compliance and the dynamic nature of gene expression require further exploration. Future research should concentrate on elucidating the molecular mechanisms underlying the core genes and refining the model to better meet clinical needs, thereby supporting precision medicine for UCEC patients.

In recent investigations concerning PCD and UCEC, Meng et al. identified a dozen PCD-related genes across 14 distinct PCD patterns, subsequently developing a prognostic model to examine the impact of PCD scores on patient outcomes (60). Similarly, Xiong et al. employed the LASSO machine learning technique on a dataset of 200 PCD-related genes, selecting 11 significant genes to construct a prognostic model for UCEC. Their analysis extended to exploring the correlations among PCD genes, immune infiltration, and drug sensitivity (61). Compared to these studies, our research demonstrates several key innovations and advantages. First, we integrated 18 PCD patterns encompassing 1,548 PCD-related genes, representing a nearly eightfold increase compared to existing studies. This expansion offers a more comprehensive understanding of the molecular landscape of PCD in UCEC. Second, we introduced five advanced machine learning algorithms and employed cross-validation to select core genes, effectively mitigating biases associated with reliance on a single algorithm and ensuring robust and reliable results. Third, we identified novel core genes—NT5E, SRPX, and ATP6V1C2—that have rarely been reported in UCEC research. This marks the first recognition of these genes as potential prognostic biomarkers, highlighting their significant clinical research value. Fourth, we extended our analysis beyond prognostic modeling to investigate TMB and immune escape mechanisms. This provides insights into immunotherapy responsiveness and lays a theoretical foundation for personalized treatment strategies. Finally, addressing the lack of experimental validation in previous studies, we validated the expression levels of core genes using RT-qPCR experiments on clinical samples, significantly enhancing the credibility and translational potential of our findings. Collectively, these innovations not only underscore the strengths of our study but also provide a more comprehensive and clinically relevant framework for understanding PCD in UCEC. Future investigations could further explore the underlying mechanisms of these core genes to facilitate their application in precision medicine for UCEC.

In the RT-qPCR experimental validation, we observed a divergence in the expression trend of NT5E when compared to the results obtained from the TCGA-UCEC dataset. Specifically, while NT5E was found to be downregulated in the TCGA-UCEC dataset, it appeared upregulated in our RT-qPCR validation. To elucidate this inconsistency, it is pertinent to note that previous research has indicated that the expression of NT5E may vary depending on the stage of the disease. In early-stage endometrial cancer, TGF-β1 has been shown to upregulate NT5E expression, which contributes to the maintenance of epithelial cell integrity and thus inhibits tumor progression. Conversely, as the tumor becomes more invasive, there is a marked reduction in NT5E expression in cases exhibiting deep invasion at stage I or in more advanced stages of endometrial cancer (46). In our study, the majority of patients in the TCGA-UCEC dataset were classified at stage IB or higher, whereas the samples used for the RT-qPCR experiments predominantly consisted of stage IA patients. This disparity in staging likely accounts for the observed discrepancies in NT5E expression results. Although a stratified analysis of the TCGA-UCEC data has not yet been conducted in this study, based on the existing literature and data, we postulate that NT5E expression may undergo dynamic changes correlating with the progression of UCEC: higher levels of expression are likely in early-stage tumors (e.g., stage IA) but tend to decrease as the disease progresses to more invasive stages (e.g., stage IB or higher). Consequently, the predominance of late-stage patients in the TCGA dataset might lead to an overall trend of NT5E downregulation, whereas the RT-qPCR validation, focusing on early-stage patients, may detect an upregulation of NT5E expression. This underscores the importance of considering tumor stage when evaluating NT5E expression. In future research, we aim to stratify the TCGA-UCEC data to examine NT5E expression across different stages and subtypes, and to expand RT-qPCR experiments to encompass a broader range of UCEC patients at varying stages. Additionally, we plan to investigate NT5E expression in specific cell types within the tumor microenvironment to enhance our understanding of its role in UCEC progression. These endeavors will elucidate the dynamic expression patterns of NT5E and fortify its potential as a prognostic biomarker.

In the external validation set, ATP6V1C2 did not exhibit significant differences in expression between the normal and cancer groups. This inconsistency could be ascribed to variations in sample origin, the technical platforms employed, or tumor heterogeneity within the dataset. Nonetheless, ATP6V1C2 demonstrated significant differential expression in the TCGA dataset and was further validated through RT-qPCR experiments, reinforcing its role as a core gene in the model. Although results from a single dataset may have limitations, the overall integrity of this study remains robust. Future research will concentrate on multicenter validation to enhance the generalizability of the model.

In conclusion, the prognostic model based on ATP6V1C2, SRPX, and NT5E demonstrates substantial predictive ability and potential for clinical application. By integrating molecular, immune, clinical features, and drug sensitivity, this model provides a scientific foundation for precise stratification management and personalized treatment for patients. However, the model requires further refinement, particularly in elucidating the underlying biological mechanisms of ATP6V1C2, SRPX, and NT5E to better comprehend their roles in tumor progression and patient prognosis. Multi-center and multi-platform validations are crucial to verify the robustness and generalizability of the model, ensuring its applicability across diverse populations and clinical settings. Additionally, future research should focus on assessing the model’s utility in clinical decision-making through prospective clinical trials and dynamic monitoring of tumor biology. Addressing these aspects will enhance the model’s reliability and pave the way for its practical application in precision oncology.

## Data Availability

Publicly available datasets were analyzed in this study. This data can be found here: https://www.cancer.gov/ccg/research/genome-sequencing/tcga.
