# Supplementary figures and images for: Construction of a prognostic model for endometrial cancer related to programmed cell death using WGCNA and machine learning algorithms
Source: Front Immunol. 2025 May 20;16:1564407. doi: 10.3389/fimmu.2025.1564407 (PMC12129963; doi:10.3389/fimmu.2025.1564407)

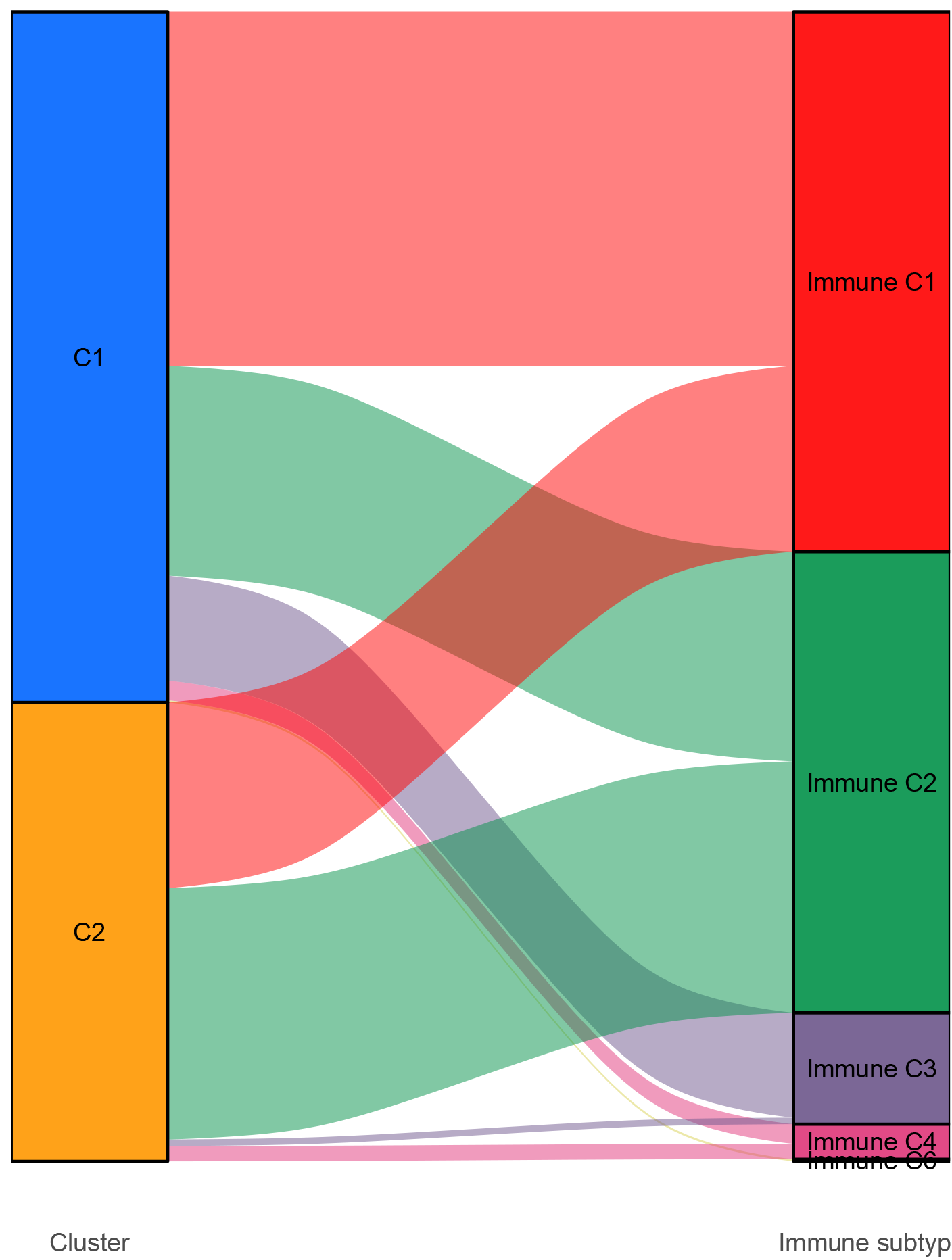

Supplement: Supplementary file 1 [file DataSheet1.zip › Supplementary materials/Supplementary Figure S1.tif]

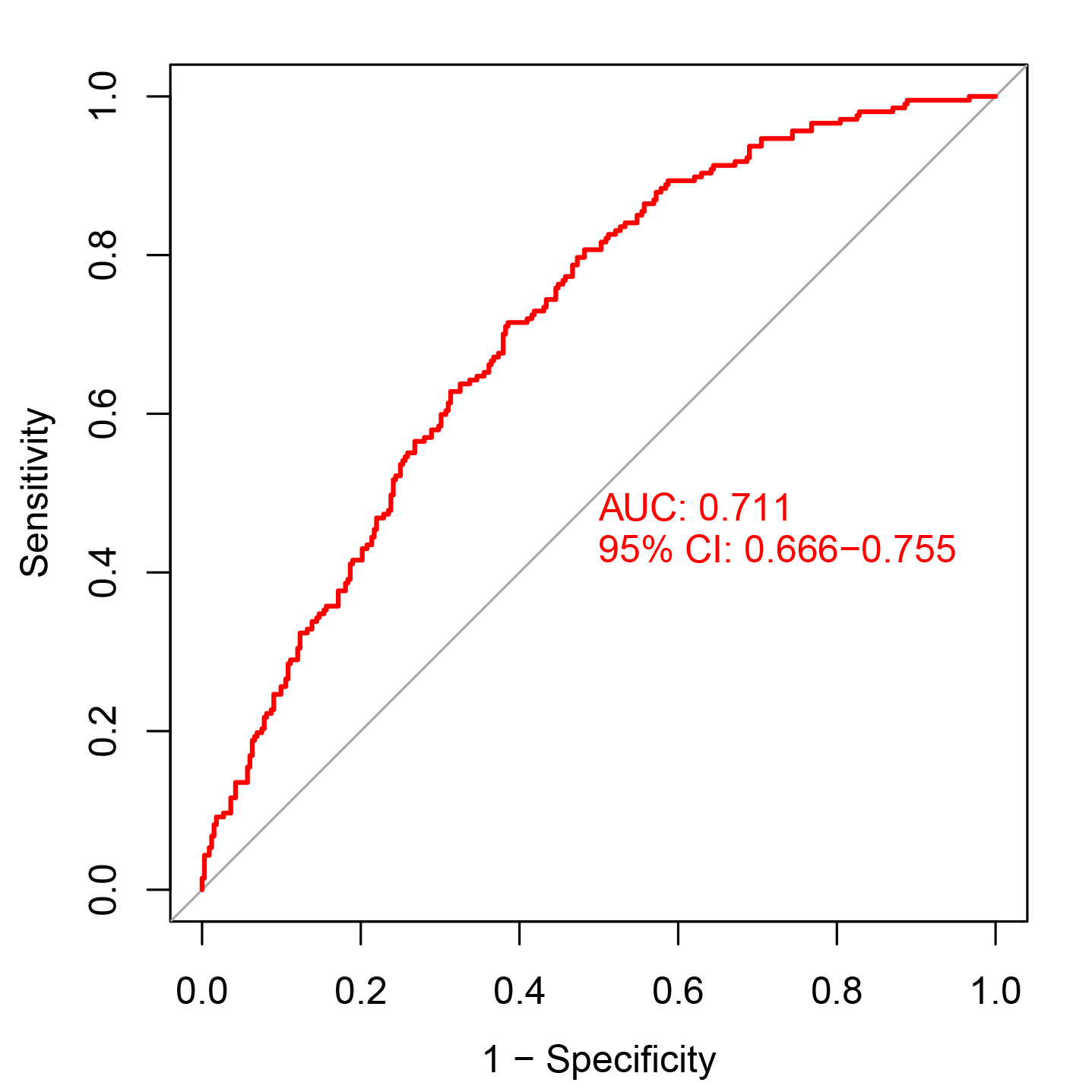

Supplement: Supplementary file 1 [file DataSheet1.zip › Supplementary materials/Supplementary Figure S3.tif]

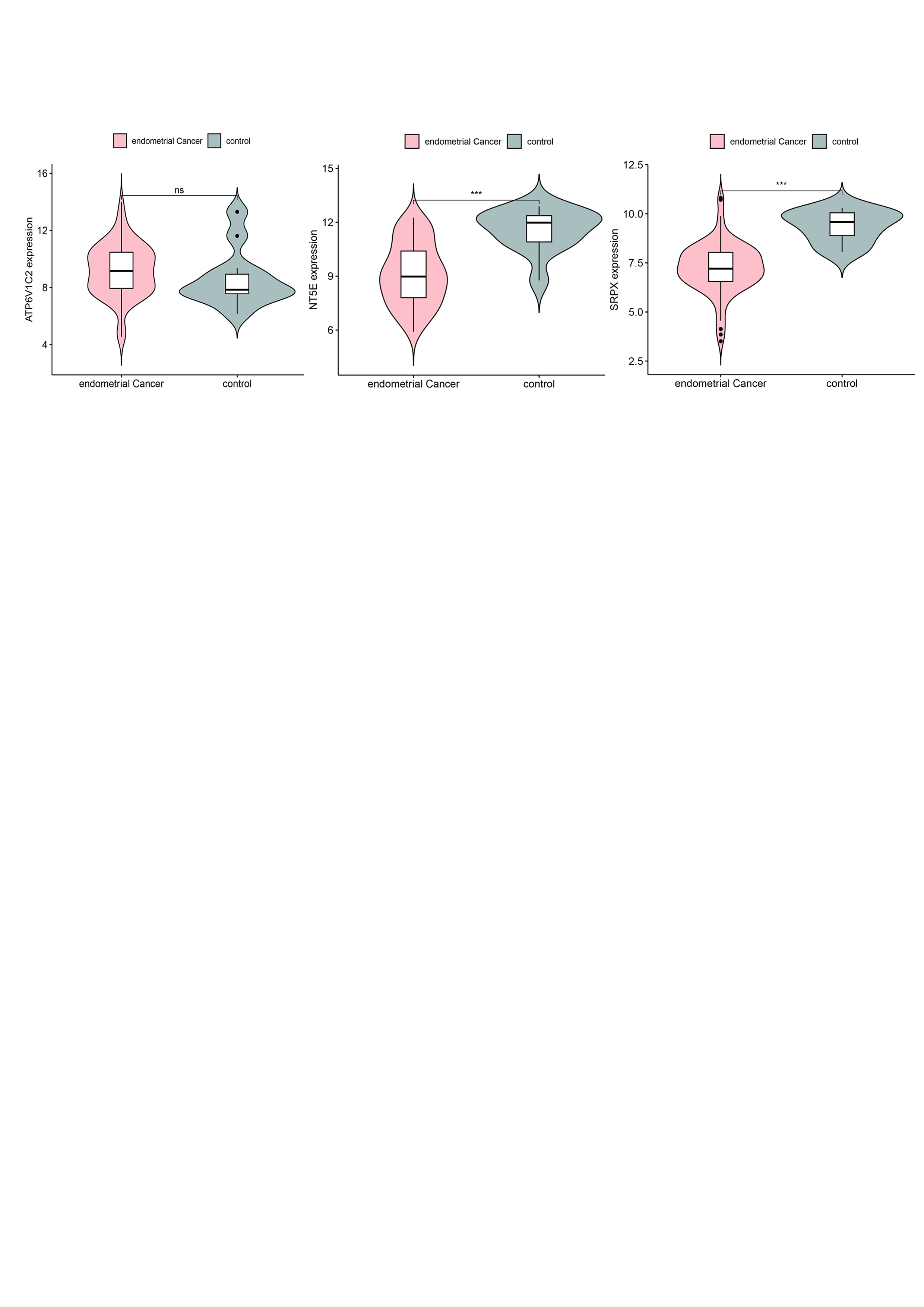

Supplement: Supplementary file 1 [file DataSheet1.zip › Supplementary materials/Supplementary Figure S4.tif]
